# The Assessment for Sensitivity of a NO_2_ Gas Sensor with ZnGa_2_O_4_/ZnO Core-Shell Nanowires—a Novel Approach

**DOI:** 10.3390/s100403057

**Published:** 2010-03-30

**Authors:** I-Cherng Chen, Shiu-Shiung Lin, Tsao-Jen Lin, Cheng-Liang Hsu, Ting Jen Hsueh, Tien-Yu Shieh

**Affiliations:** 1 Micro Systems Technology Center, ITRI South, Industrial Technology Research Institute, Tainan 709, Taiwan; 2 College of Dental Medicine, Kaohsiung Medical University, Kaohsiung 807, Taiwan; 3 Chemical Engineering Department, National Chung-Cheng University, Chia-Yi, 621, Taiwan; 4 Department of Electronic Engineering National University of Tainan, Tainan, 700, Taiwan; 5 National Nano Device Laboratories, Tainan 741, Taiwan

**Keywords:** ZnGa_2_O_4_, ZnO, core shell, nanowire, NO_2_, gas sensor, sensitivity

## Abstract

The application of novel core-shell nanowires composed of ZnGa_2_O_4_/ZnO to improve the sensitivity of NO_2_ gas sensors is demonstrated in this study. The growth of ZnGa_2_O_4_/ZnO core-shell nanowires is performed by reactive evaporation on patterned ZnO:Ga/SiO_2_/Si templates at 600 °C. This is to form the homogeneous structure of the sensors investigated in this report to assess their sensitivity in terms of NO_2_ detection. These novel NO_2_ gas sensors were evaluated at working temperatures of 25 °C and at 250 °C, respectively. The result reveals the ZnGa_2_O_4_/ZnO core-shell nanowires present a good linear relationship (R^2^ > 0.99) between sensitivity and NO_2_ concentration at both working temperatures. These core-shell nanowire sensors also possess the highest response (<90 s) and recovery (<120 s) values with greater repeatability seen for NO_2_ sensors at room temperature, unlike traditional sensors that only work effectively at much higher temperatures. The data in this study indicates the newly-developed ZnGa_2_O_4_/ZnO core-shell nanowire based sensors are highly promising for industrial applications.

## 1. Introduction

Nitrogen dioxide (NO_2_) is a toxic compound with a pungent odor that is harmful to the environment as a major cause of acid rain and photochemical smog. NO_2_ is mainly produced by power plants, combustion engines and automobiles. It can also be noxious and induce health problems, such as olfactory paralysis. Safety guidelines recommend that humans should not be exposed to more than 3 ppm NO_2_ gas for periods longer than 8 hours [1–3]. Therefore, it is highly desirable to develop a reliable sensor that can effectively detect NO_2_ even with extremely low concentration.

An ideal sensor can be utilized in an early warning system for environmental monitoring to detect the presence of NO_2_ before a critical condition occurs. The demand to produce ideal NO_2_ sensors has propelled considerable research activities in the relevant fields. Various types of metal oxide based sensors composed of TiO_2_, SnO_2_, ZnO, or WO_3_ have been used extensively to detect toxic and pollutant gases, such as NO_x_, H_2_S, Cl_2_, CO, SO_2_, and O_3_. Other combustible gases, including H_2_, CH_4_ and flammable organic vapors are also detectable by these compound based sensors [4–6]. Recently, numerous compounds have been applied for NO_2_ detection, and YSZ, NASICON, ZnO, In_2_O_3_, and WO_3_ have been extensively investigated [7–19]. Among them, ZnO is a chemically and thermally stable n-type semiconductor which is highly advantageous for the development of the ideal sensors. It incorporates both massive exciton binding energy (60 *MeV*) and significant band-gap energy (3.37 *eV*) at room temperature [20]. ZnO subsequently is frequently applied for generating novel detectors, particularly for NO_2_ [21]. ZnO gas sensors were widely demonstrated in the forms of thick films, thin films, heterojunctions, nanoparticles, and nanowires [10,11,22–28]. It is also noted that the use of semiconductor metal oxide materials for gas sensing generally involves some chemical reaction with gas molecules on the oxide surface [29].

Nowadays one-dimensional ZnO nanowires are drawing considerable attention for their larger surface-to-volume ratio than bulk ZnO and ZnO films [30]. Due to its larger surface area, the nanowire-based gas sensors tend to provide much higher sensitivity. Laboratory manipulations and manufacture of ZnO nanowires have been reported in our previous studies. These include vertical growth of ZnO nanowires on various substrates without catalyst [31–34], introduction of dopants into the ZnO nanowires [35,36], and production of vertical core–shell nanowires grown of ZnGa_2_O_4_/ZnO by applying a low-pressure chemical vapor transport [37,38]

ZnGa_2_O_4_ is an attractive low-voltage phosphor [39,40]. It is a transparent conducting oxide with a wide band-gap and a spinel crystal structure [41,42]. The structure of ZnGa_2_O_4_/ZnO core-shell nanowires uniquely consists of a ZnO core and a thin layer of spinel ZnGa_2_O_4_ shell. Its use in gas sensors has not been formally reported, despite its advantageous characteristics. It is well known that spinel oxides (AB_2_O_4_) can be used as gas sensor materials because of their stability to thermal and chemical conditions.

It is proposed in this study to apply ZnGa_2_O_4_/ZnO core-shell nanowires as a novel sensor for NO_2_ detection. It is generated on patterned ZnO:Ga/SiO_2_/Si templates at 600 °C by reactive evaporation to form a form simple sensing structure. This research reports the manufacturing procedures and its chemical sensing properties for NO_2_ tracing. The potential industrial application of ZnGa_2_O_4_/ZnO core-shell nanowires is also discussed.

## 2. Materials and Methods

### 2.1. Preparation of Chemicals and Materials

p-Type (100) oriented Si wafers (Si Wave Co., Taiwan) were used as the substrates. They were initially cleaned by an RCA standard process to remove organic contaminants, and then rinsed under running de-ionized water. To thoroughly remove native oxide, the wafers were dipped into 48% HF solution for 10 seconds, and flushed by dry nitrogen. The chemicals used in this study included Zn metal powder (99.9%, 300 mesh, Strem Chemicals, Newburyport, MA, USA), Gallium powder (99.99%, 100 mesh, American Elements Products), O_2_ (99.999%, Air Products, Taiwan), N_2_ (99.99%, Air Products, Taiwan) and Ar (99.999%, Air Products, Taiwan). The target of ZnO:Ga with a mixture of ZnO (99.99% Strem Chemicals) and Ga_2_O_3_ (99.999%, Strem Chemicals) was employed as source materials. The target was prepared using conventional sintering process. The amount of Ga_2_O_3_ added to the target was 3 wt%. All aqueous solutions were prepared with purified water obtained from the Ropure ST water purification system (Barnstead) with a specific resistance of 18 Mohm-cm.

### 2.2. Preparation of ZnGa_2_O_4_/ZnO Core-Shell Nanowire Based Sensors

**Figure 1.**
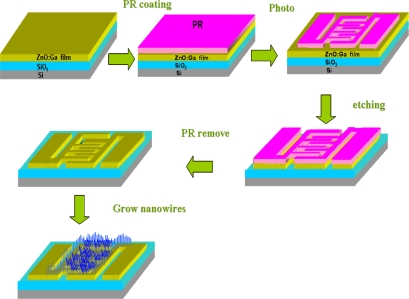
The growth and processing steps of ZnGa_2_O_4_/ZnO core-shell nanowire based sensors.

Figure 1 schematically depicts the growth and processing steps used in this study. Prior to growing the ZnGa_2_O_4_/ZnO core-shell nanowires, a cleaned Si (100) substrate was thermally oxidized to form a 500 nm-thick SiO_2_ film. A 100 nm-thick Ga-doped ZnO thin film was subsequently deposited onto the SiO_2_ film by RF magnetron sputtering. X-ray diffraction (XRD) measurement showed that the sputtered ZnO:Ga film was oriented along the (0 0 2) direction. The sheet resistance of the sputtered ZnO:Ga film was evaluated by four-point resistivity measurement, and it was found to be around 200 Ω/sq. Afterwards, a comb-like pattern was formed by partially etching away the ZnO:Ga film with standard photolithography methods. During wet etching, the template was dipped in 2% HCl for 3 min to remove the exposed ZnO:Ga. Through the designed etching mask, the fingers of the comb-like pattern were developed in 10 μm wide and 80 μm long with a spacing of 10 μm, as shown in Figure 1. Subsequently, two small pieces of glasses were used to cover the two electrodes of the patterned ZnO:Ga film to prevent growing ZnGa_2_O_4_/ZnO core-shell nanowires in these regions.

To grow the ZnGa_2_O_4_/ZnO core-shell nanowires, the patterned ZnO:Ga/SiO_2_/Si substrate and Zn/Ga mixture powder were placed in an alumina boat, and then the alumina boat inserted into a quartz tube. Zinc metal powder with 99.9% purity was applied as the Zn source, and the Ga source was 99.99% pure Ga powder. Before growth, Zn and Ga ingredients were prepared by grinding these powders together into fine mixtures with ratio of 0.3 g/0.15 g.

Constant streams of argon (54.4 sccm) and oxygen (0.8 sccm) gases were then introduced into the furnace. The critical positions and processing temperature of the patterned ZnO:Ga/SiO_2_/Si substrate, Zn/Ga vapor source and alumina boat were carefully controlled. They have to be located at the same horizontal level and heated at the same temperature. A mechanical pump was subsequently employed to evacuate the system, and a programmable temperature controller was used to precisely control the furnace temperature with an accuracy of ±1 °C. During the growth of nanowires, the quartz tube pressure and the growth temperature were maintained at 10 Torr and 600 °C, respectively. The process lasted 60 minutes. For comparison purposes, pure ZnO nanowires without any Ga were also prepared under exactly the same conditions.

### 2.3. Characterization of ZnGa_2_O_4_/ZnO Core-Shell Nanowires

A MAC MXP18 X-ray diffractometer and a JEOL JEM-2100F high resolution transmission electron microscopy (HRTEM), operated at 200 KV, were then used to characterize the crystallography and structure of the as-grown nanowires. The surface morphologies of the samples and the size distribution of the nanowires were characterized using a JEOL JSM-6500F field emission scanning electron microscope (FESEM), operated at 5 KeV. Photoluminescence (PL) properties of the as-grown ZnO nanowires were also characterized by a Jobin Yvon-Spex fluorolog-3 spectrophotometer. A Xe lamp emitting at 254 nm was applied as the excitation source during PL measurements.

### 2.4. Measurement of Gas Sensing Properties

To measure gas sensing properties of the nanowires, the sample was prepared in a sealed glass chamber. Its resistivity in air was measured from the two electrodes of the patterned ZnO:Ga film. In this study, NO_2_ gas was used as the target for detection. NO_2_ gas was carried by air or N_2_ into the glass chamber through a mixer. Gas-sensing tests were all performed at room temperature. The total flow rate of the NO_2_ and the carrier gas was kept constantly at 100 cm^3^/min in each test. In order to control temperature, a small heater equipped with a thermocouple was used. The screen printing method was utilized to produce the heating plate, by using the RuO_2_ paste on alumina substrate with silver-printed electrodes (area: 10 mm × 10 mm; heater resistance: 30 ohm). Manipulation of NO_2_ concentrations was carried out by modulating the ratio of the flow rate of NO_2_ gas to that of the carrier gas. The electrical response of the sensor was measured with a computer-loaded analytic system. A voltage detecting method was used to calculate the sensitivity of the sensor, and it was defined as:

(1) $$S=(R_{s}-R_{\text{air}})/R_{\text{air}}$$

where S represents sensitivity, R_s_ and R_air_ were the electrical resistances in NO_2_ and synthetic air, respectively. To observe dynamic and repetitive responses, the sensor was fixed on a temperature-controlled heater, and placed inside a 100-mL glass chamber. The testing gas mixture was continuously flowing into the glass chamber. The flow rate was kept constantly at 100 cm^3^/min, and NO_2_ concentrations in synthetic air were varied from 1–100 ppm. Pure ZnO nanowires without any Ga were also prepared and tested in the same way as the control group.

## 3. Results and Discussion

### 3.1. Morphological and Electronic Properties of ZnGa_2_O_4_/ZnO Core-Shell Nanowires

**Figure 2.**
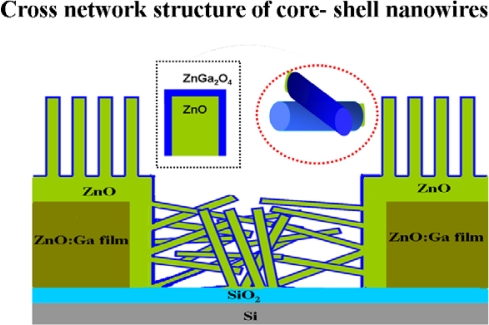
A schematic diagram of cross network structure as sensing layers of the ZnGa_2_O_4_/ZnO core-shell nanowires based sensors.

**Figure 3.**
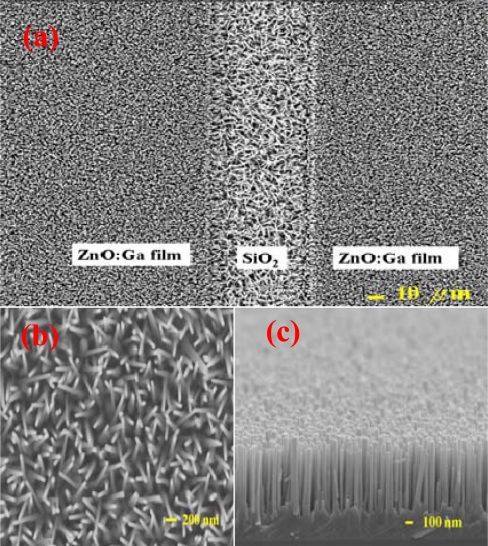

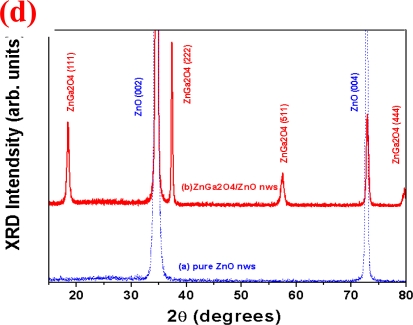
(a) FESEM image of ZnGa_2_O_4_/ZnO core-shell nanowires grown on patterned ZnO:Ga/SiO_2_/Si substrate. (b) Enlarged SEM photographs of ZnGa_2_O_4_/ZnO core-shell nanowires grown on the SiO_2_ spacer regions (c) Tilted cross sectional FESEM images of ZnGa_2_O_4_/ZnO core-shell nanowires grown on the ZnO:Ga film regions the sample (d) XRD spectrum of pure ZnO nanowires (a-blue-line) and ZnGa_2_O_4_/ZnO core-shell nanowires (b-red-line) prepared in this study.

Figure 2 shows the schematic illustration for a cross network of ZnGa_2_O_4_/ZnO core-shell nanowires floating on a patterned ZnO:Ga/SiO_2_/Si substrate. The structure of ZnGa_2_O_4_/ZnO core-shell nanowires consists of a ZnO core and a thin layer of ZnGa_2_O_4_ shell. These grow on conductive film of ZnO:Ga in the vertical and orderly shape, compared with cross network structure of growth on SiO_2_ insulation of spacer regions, which constitutes a simple gas sensor. A simple and efficient way is presented in this study to produce the ZnGa_2_O_4_/ZnO core-shell based gas sensors with self-assembly. This process is inexpensive and feasible for nano-devices, and it is illustrated as below:

Figure 3(a) shows a top view FESEM image of the as-grown ZnGa_2_O_4_/ZnO core-shell nanowires prepared on the patterned ZnO:Ga/SiO_2_/Si template. It was found that the ZnGa_2_O_4_/ZnO nanowires were grown vertically on the conducting ZnO:Ga finger regions. This should be attributed to the fact that these ZnGa_2_O_4_/ZnO core-shell nanowires were grown along the columnar grains of the underneath sputtered ZnO:Ga film [43]. In contrast, the growing alignment of ZnGa_2_O_4_/ZnO core-shell nanowires grown on SiO_2_ spacer regions in the inset of Figure 3(b) was randomly oriented. These randomly aligned ZnGa_2_O_4_/ZnO core-shell nanowires tend to provide electrical paths between the adjacent fingers. Figure 3(c) indicates the titled cross sectional FESEM images of the ZnGa_2_O_4_/ZnO core-shell nanowires vertical grown on the ZnO:Ga film regions the sample. Figure 3(d) demonstrated the X-ray diffraction (XRD) spectrum of the pure ZnO nanowires (*a-blue*-line) and the ZnGa_2_O_4_/ZnO core-shell nanowires (*b-red*-line), and this was prepared to characterize the structural properties.) In addition to ZnO-related peaks, the XRD spectrum in Figure 3(d) demonstrated ZnGa_2_O_4_ (111), (222), (511) and (444) peaks. This observation reveals that the nanowires possess a ZnGa_2_O_4_ crystal structure. The analysis of FESEM images show that the as-grown ZnGa_2_O_4_/ZnO nanowires present a core-shell structure with 0.8–5 μm in length and 40–100 nm in width. These core-shell nanowires, consisting of a ZnO core and a thin layer of spinel ZnGa_2_O_4_ shell, have not been investigated in terms of its efficacy of gas detection. It is therefore proposed to thoroughly inspect its NO_2_ gas sensing mechanisms.

### 3.2. Efficacy and Sensitivity of Gas Detection of ZnGa2O4/ZnO Nanowires

For NO_2_ gas sensing, oxygen sorption plays an important role in electrical transport properties of ZnO nanowires. The oxygen ionosorption removes conduction electrons and thus lowers the conductance of ZnO [44]. The reactive oxygen species such as O_2_^−^, O^2−^ and O^−^ are first adsorbed on ZnO surface when temperature rises. It is well known that the response of chemisorbed oxygen species strongly depends on temperature. At low temperatures, O_2_^−^ is commonly chemisorbed. At high temperatures, however, O^−^ and O^2−^ are chemisorbed while O_2_^−^ disappears [45]. The reaction kinematics can be described as follows [44]:

(2) $$O_{2\;(\text{gas})}↔O_{2\;(\text{ads})}$$

(3) $$O_{2\;(\text{ads})}+e^{-}↔O_{2}{}^{-}$$

(4) $$O_{2}{}^{-}+e^{-}↔2\;O^{-}$$

When the ZnO nanowires are exposed to NO_2_ gas, NO_2_ gas tends to react with the adsorbed O^−^ ions and directly accumulate on the surface of ZnO nanowires. And its reactions are shown as below [47,48]:

(5) $${\text{NO}}_{2\;(\text{gas})}+e^{-}\to {{{\text{NO}}_{2}}^{-}}_{\left(\text{ads}\right)}$$

(6) $${\text{NO}}_{2}{{}^{-}}_{\left(\text{ads}\right)}+O^{-}{}_{\left(\text{ads}\right)}+2e^{-}\to {\text{NO}}_{\left(\text{gas}\right)}+2O^{2-}{}_{\left(\text{ads}\right)}$$

**Figure 4.**
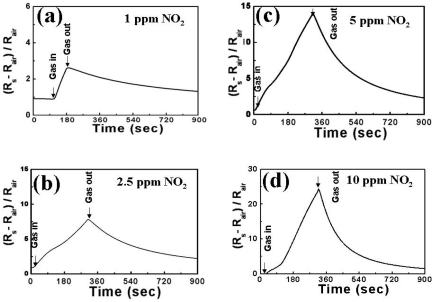
Dynamic responses of ZnGa_2_O_4_/ZnO core-shell nanowire sensor to different NO_2_ concentrations of (a) 1 ppm, (b) 2.5 ppm, (c) 5 ppm, and (d) 10 ppm in synthetic air at 250 °C, respectively.

Subsequently, the concentration of electrons on the surface of ZnO nanowires arrays decreases and the resistance of ZnO layer will increase accordingly. The adsorption of O^−^ ions is an very interesting and critical phenomenon in metal-oxide gas sensor, because the O^−^ ions tend to assist the adsorbed NO_2_^−^ ions in taking the electrons from the nanowires arrays. The dynamic responses of ZnGa_2_O_4_/ZnO core-shell nanowires sensors at 250 °C were tested with four different NO_2_ concentrations at 1, 2.5, 5 and 10 ppm, respectively (Figure 4). The gas-input period of NO_2_ gas was varied from 90 to 360 seconds.

A complete recovery was observed after the regeneration process in dry air. Since NO_2_ gas does not generate any poisoning effects on the sensors, it is very likely that its total recovery is highly attainable. This investigation also reveals that the latent period between each response of gas detection at 250 °C was much longer than we expected to observe steady-state. It is therefore proposed to apply 360 s between each test in order to standardize the procedure of signal recorded.

**Figure 5.**
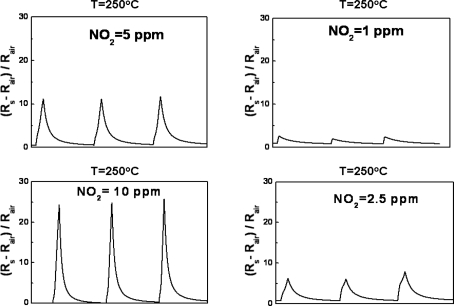
Repetitive response curves of ZnGa_2_O_4_/ZnO core-shell nanowires sensors operated at 250 °C. The different concentrations of testing gas are 1 ppm, 2.5 ppm, 5 ppm, and 10 ppm NO_2_ in N_2_ carrier gas.

The result shown in Figure 5 is the sensitivity of the ZnGa_2_O_4_/ZnO core-shell nanowires sensors in response to repetitive adsorption–desorption cycles. The stability and reproducibility of the NO_2_ sensors at 250 °C were demonstrated with four different NO_2_ concentrations in 1, 2, 5 and 10 ppm, respectively.

**Figure 6.**
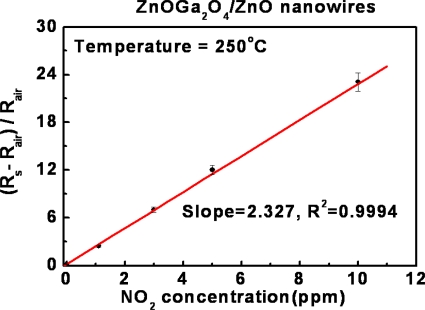
Response calibration of ZnGa_2_O_4_/ZnO core-shell nanowire sensors for different NO_2_ concentrations at 250 °C.

Figure 6 shows the relative response of the sensor is linearly proportional to NO_2_ concentration ranged from 1 to 10 ppm at 250 °C. The sensitivity can be calculated from the slope as 2.327 ppm^−1^ and the quality of the curve fit as R^2^ = 0.9994. There are many nanowire-to-nanowire junctions at the networking points of ZnGa_2_O_4_/ZnO core-shell nanowires based sensor. Thus, the enhanced sensitivity of the ZnGa_2_O_4_/ZnO core-shell nanowires can be attributed to the changes in the resistance of the gas sensors due to both a surface depletion region of each nanowire and the potential barrier height in the junction.

**Figure 7.**
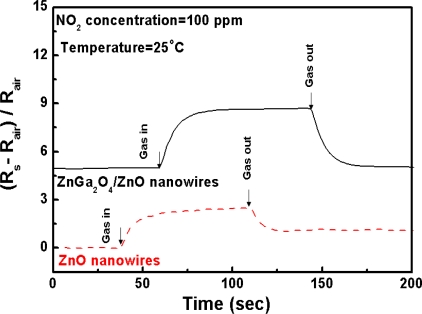
Dynamic responses of ZnGa_2_O_4_/ZnO core shell nanowire sensor and ZnO nanowire sensor to 100 ppm NO_2_ concentration in synthetic air at 25 °C.

**Figure 8.**
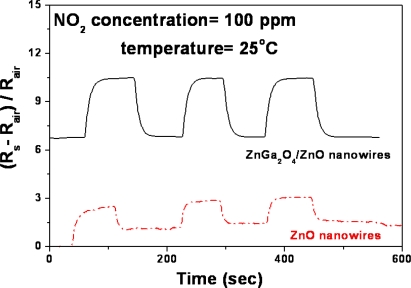
Repetitive response curves of ZnGa_2_O_4_/ZnO core-shell nanowire sensor and ZnO nanowire sensor at 25 °C, respectively. The testing gas is 100 ppm NO_2_ in N_2_ carrier gas.

Figures 7 and 8 show the sensitivity of ZnGa_2_O_4_/ZnO core-shell nanowires sensor and ZnO nanowires sensor in response to repetitive adsorption–desorption cycles. The stability and reproducibility of the NO_2_ sensor is demonstrated at 25 °C. The testing gas is 100 ppm NO_2_ in N_2_ carrier gas. The room temperature sensitivity observed here is most likely to be due to the high surface-to-volume ratio of the one-dimensional nanostructures. Meanwhile, since ZnGa_2_O_4_/ZnO core-shell nanowires are an n-type semiconductor, the oxidizing NO_2_ molecules adsorbed on the oxide surface may capture electrons from the conduction band and form NO_2_ [49]. This micro-property tends to increase the carrier concentration and leads to a greatly reduced resistivity of the nanowires. In our study [38], the electrical properties of ZnGa_2_O_4_/ZnO core-shell nanowires have been assessed by Hall-effect measurements in the previous study [39]. The outcome showed that the values of conductivity and carrier concentration are 33 S/cm and 1.02 × 10^22^ cm^−3^, respectively. These are much higher than those of the pure ZnO nanowires (0.8 S/cm and 4 × 10^16^ cm^−3^, respectively). The comparison of the data indicates the ZnGa_2_O_4_/ZnO core-shell nanowires are superior to ZnO nanowires in electric characteristics. The former is 40 times the conductivity, and the 2.55 × 10^5^ times the carrier concentration of the latter. This explains the advantage of ZnGa_2_O_4_/ZnO core- shell nanowires for NO_2_ sensing. Compared with the ZnO nanowires, the electric characteristic of ZnGa_2_O_4_/ZnO core-shell nanowires is 40 times higher on conductivity and 2.55 × 10^5^ times higher on carrier concentration. For this reason we prefer using ZnGa_2_O_4_/ZnO core-shell nanowires for the NO_2_ sensing.

**Figure 9.**
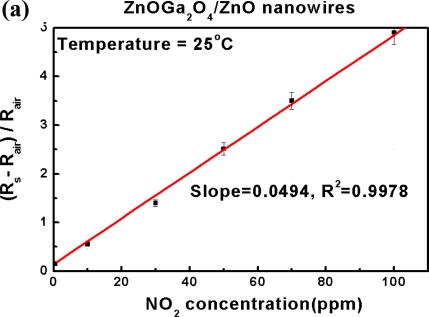

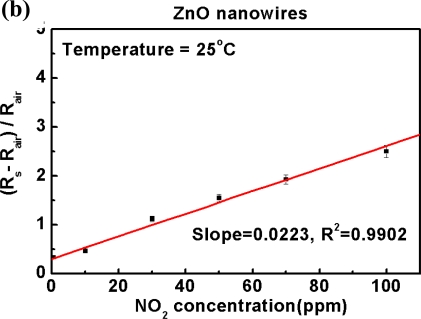
Response calibrations of **(a)** ZnGa_2_O_4_/ZnO core-shell nanowire sensor and **(b)** pure ZnO nanowire sensor for different NO_2_ concentrations at 25 °C.

Figure 9 shows the relative response of the sensor is linearly proportional to different NO_2_ concentrations ranged from 10 to 100 ppm at 25 °C. The sensitivity of ZnGa_2_O_4_/ZnO core-shell nanowire sensors calculated from the slope was 0.0494 ppm^−1^, while the ZnO nanowire sensors only presents its sensitivity as 0.0223 ppm^−1^.

The calculation of slope can be used as an index of sensor sensitivity, and it is obvious that the slope of ZnGa_2_O_4_/ZnO core-shell nanowires is greater than twice that of of the ZnO nanowires. This demonstrates that the sensitivity of NO_2_ gas detection by the ZnGa_2_O_4_/ZnO core-shell nanowires can be significantly enhanced at room temperature. Both tests reveal good linear relationships (R^2^ > 0.99) between sensitivity and gas concentration. The response time to detect 100 ppm NO_2_ is about 90 seconds to reach a stable state, and recovery time back to the background is about 120 seconds at room temperature. In contrast, the sensitivity of the ZnGa_2_O_4_/ZnO core-shell nanowires at 250 °C is much greater than that at 25 °C, but the response time is too long to reach stability. The results show that the ZnGa_2_O_4_/ZnO core-shell nanowires based sensors possess the best response and recovery with greater repeatability for NO_2_ sensors occurred at room temperature.

All the metal oxide sensors inevitably have the problem of selectivity in practical NO_2_ detection applications, especially in the presence of reducing gases, such as H_2_, CO, VOCs. Most gases sensors including those for VOCs and EtOH can only work properly at much higher temperatures above 200°C. It is therefore a great demand for NO_2_ sensors working effectively at ordinary temperatures so that the interferences can be substantially reduced. At room temperature, the ZnGa_2_O_4_/ZnO core-shell nanowires based sensors as NO_2_ sensors present the following characteristics: (1) good linear relationship between sensitivity and NO_2_ concentrations, (2) the utmost response (<90 s) and (3) a useful recovery period (<120 s) with greater repeatability for NO_2_ detection. In the future work of this study will be extended for improving selective and sensitivity with low-temperature operation. For expansion of its potential application, the further investigation in terms of specificities and sensitivities on other types of gas detection will be conducted in low-temperature settings.

## 4. Conclusions

This investigation reports on the growth of ZnGa_2_O_4_/ZnO core-shell nanowires on patterned ZnO:Ga/SiO_2_/Si templates and the fabrication of the NO_2_ gas sensors based on these ZnGa_2_O_4_/ZnO core-shell nanowires. The ZnGa_2_O_4_/ZnO core-shell nanowires grown on a sputtered ZnO:Ga layer were vertically aligned while those grown directly on the SiO_2_ layer were randomly oriented, to form the simple sensors with homogenized nanostructure. The ZnGa_2_O_4_/ZnO core-shell nanowires demonstrate good linear relationship between sensitivity and NO_2_ concentration both at 250 °C and 25 °C. The NO_2_ gas detection of the ZnGa_2_O_4_/ZnO core-shell nanowires sensors present its best response (<90 s) and recovery period (<120 s) with greater repeatability at room temperature. The results indicate that the developed ZnGa_2_O_4_/ZnO core-shell nanowires based sensors are highly promising for industrial applications.
